# Preoperative prediction of p53 overexpression in pituitary neuroendocrine tumors using MRI radiomics

**DOI:** 10.3389/fneur.2025.1693959

**Published:** 2026-01-23

**Authors:** Longyuan Gu, Fanghua Zhou, Bin Wu, Jianpin Yang, Bin Li, Yuechao Fan, Peizhi Ji, Qian Wu, Fengda Li, Shuhong Mei

**Affiliations:** 1 Department of Neurosurgery, Ji’an Central People’s Hospital, Ji’an, Jiangxi, China; 2 Department of Operating Room, Ji’an Central People’s Hospital, Ji'an, Jiangxi, China; 3 Department of Neurosurgery, The Affiliated Hospital of Xuzhou Medical University, Xuzhou, Jiangsu, China; 4 Department of Ultrasound Medicine, Ji’an Central People’s Hospital, Ji'an, Jiangxi, China; 5 Department of Neurosurgery, Changshu Hospital Affiliated to Soochow University, Changshu, China

**Keywords:** MRI radiomics, nomogram, p53 overexpression, pituitary neuroendocrine tumors, preoperative prediction model

## Abstract

**Background:**

The expression of p53 protein is closely related to tumor prognosis and plays an important role in patients with pituitary neuroendocrine tumors (PitNETs). However, its evaluation currently relies on postoperative histopathological analysis. Developing a non-invasive method to predict p53 overexpression preoperatively may help support clinical judgment and facilitate individualized treatment strategies.

**Methods:**

Clinical and imaging data from 186 patients with pathologically confirmed PitNETs were retrospectively collected. The cohort was divided into training and testing sets using stratified random sampling. Radiomic features were extracted from MRI sequences, and feature selection was performed using the intraclass correlation coefficient (ICC) and least absolute shrinkage and selection operator (LASSO). A radiomics score was calculated, and univariate and multivariate logistic regression analyses were used to identify independent clinical risk factors. A combined nomogram model incorporating clinical and radiomic features was constructed. Model performance was assessed using the area under the receiver operating characteristic curve (AUC), precision-recall (PR) curve, calibration curve, and decision curve analysis (DCA).

**Results:**

Four radiomic features and two clinical features were selected for model development. Age (odds ratio [OR] = 0.97, 95% confidence interval [CI]: 0.94–0.99, *p* = 0.01) and suprasellar invasion (OR = 0.47, 95% CI: 0.25–0.89, *p* = 0.02) were identified as independent predictors of p53 positivity. The combined clinical-radiomic model achieved good predictive performance with an AUC of 0.77 in the validation set, demonstrating favorable discrimination, calibration, and clinical utility.

**Conclusion:**

The proposed MRI-based radiomics model, integrating clinical and imaging features, enables non-invasive preoperative prediction of p53 overexpression in PitNETs. This approach offers a promising tool for individualized risk stratification and personalized treatment planning in neurosurgical practice.

## Introduction

Pituitary neuroendocrine tumors (PitNETs), formerly referred to as pituitary adenomas (PAs), are among the most common intracranial neoplasms of the central nervous system, accounting for approximately 10–15% of all intracranial tumors (1–3). Although most PitNETs grow slowly and have favorable prognoses, some exhibit aggressive growth, are prone to recurrence, and are associated with poor clinical outcomes (4–6). To better assess the biological behavior of PitNETs, researchers have increasingly focused on molecular markers closely related to tumor progression. Among these, the p53 protein, a critical tumor suppressor, has garnered significant attention (7–9).

The p53 protein plays a crucial role in regulating the cell cycle, promoting DNA damage repair, and inducing apoptosis (10, 11). Aberrant overexpression of p53 has been observed in various tumor types and is closely associated with tumor aggressiveness, recurrence risk, and prognosis (12–17). In the context of PitNETs, studies have shown that p53 overexpression is linked to malignant transformation and aggressive tumor growth (18). Therefore, detecting p53 overexpression is of great significance for predicting the clinical behavior of PitNETs (19). However, traditional p53 detection relies on immunohistochemical analysis of postoperative tissue samples. Although accurate, this invasive method can only be performed after surgery, limiting its value for preoperative clinical decision-making.

In recent years, with the rapid advancement of medical imaging and computer analysis technologies, radiomics has emerged as a novel non-invasive diagnostic tool (20). Radiomics extracts large amounts of quantitative features from conventional medical images (e.g., MRI, CT), capturing tumor heterogeneity and underlying molecular characteristics (21–23). Compared to traditional imaging diagnostics, radiomics goes beyond visible abnormalities, uncovering latent information embedded within the images. In the case of PitNETs, MRI, as the primary imaging modality, provides a rich source of data for radiomics analysis (24, 25).

The aim of this study is to develop and validate an MRI-based radiomics model for the preoperative prediction of p53 overexpression in patients with PitNETs. As p53 overexpression has been associated with increased tumor aggressiveness, recurrence, and poor prognosis, preoperative assessment of p53 status may guide clinical decision-making, including surgical extent, postoperative surveillance strategies, and consideration of adjunctive therapies. By providing a non-invasive prediction approach, this model has the potential to assist clinicians in early identification of high-risk patients and support personalized management.

## Materials and methods

### Study subjects

**Figure 1:**
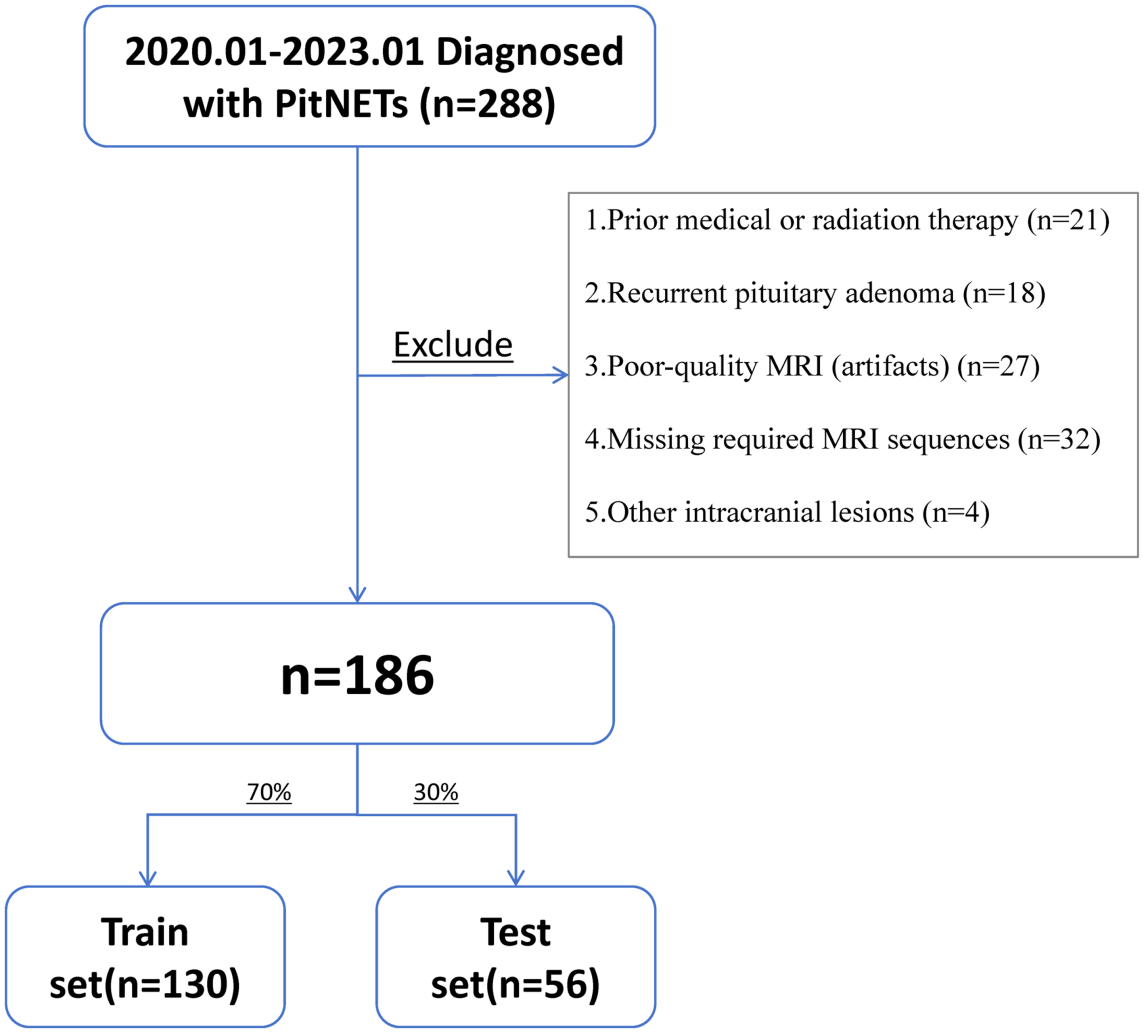
Flowchart of patient selection.

This retrospective study included 186 out of 288 patients diagnosed with PitNETs and treated at The Affiliated Hospital of Xuzhou Medical University between January 2020 and January 2023. The inclusion criteria were as follows: (1) patients underwent preoperative cranial MRI; (2) postoperative pathology confirmed the diagnosis of PitNETs; (3) immunohistochemical analysis was performed postoperatively to assess p53 expression. The exclusion criteria were: (1) a history of preoperative medical therapy or radiotherapy that may influence tumor imaging characteristics; (2) recurrent PitNETs confirmed by prior clinical or radiological records; (3) poor-quality MRI images with significant artifacts or distortions that impeded accurate delineation of the region of interest (ROI); (4) incomplete or missing imaging sequences required for radiomics feature extraction; (5) coexistence of other intracranial lesions (e.g., hemorrhage, infection, or tumor) that could interfere with image interpretation or model development. A Flowchart summarizing the screening and exclusion process is presented in Figure 1.

A total of 102 patients were excluded based on these criteria. This study was approved by the Ethics Committee of Xuzhou Medical University (Approval No. XYFY2023-KL250-01), with informed consent waived. The methods were carried out in accordance with the relevant guidelines and regulations.

### MRI data acquisition

All patients underwent scanning using a 3.0-Tesla MRI scanner (Signa HD, GE Healthcare, Waukesha, WI, United States) with an 8-channel head coil. Brain MRI scans included the following sequences: axial T1-weighted imaging (T1WI), axial T2-weighted imaging (T2WI), and enhanced T1WI sequences in axial, coronal, and sagittal planes. The contrast agent used for enhancement was gadopentetate dimeglumine (Gd-DTPA, Bayer Pharma, Germany) at a dose of 0.2 mmol/kg, with an injection rate of 3.0 mL/s. To minimize head motion during scanning, patients’ heads were stabilized using comfortable foam pads, and earplugs were provided to reduce noise from the MRI machine. The parameters for the 3.0-T MRI scans were as follows: T1WI: repetition time (TR) = 200 ms, echo time (TE) = 15 ms, T2WI: TR = 4,300 ms, TE = 120 ms, Enhanced T1WI (axial, sagittal, and coronal): same parameters as the unenhanced T1WI, Field of view (FOV): 240 mm × 240 mm, Number of slices: 18, Slice thickness: 6.0 mm, Slice gap: 1.0 mm, Number of excitations (NEX): 2.0, Matrix size: 256 × 256. All data were stored in DICOM format. A slice thickness of 6.0 mm was used based on routine clinical protocols and the need to balance signal-to-noise ratio and scan acquisition time (26). Importantly, this setting aligns with commonly adopted parameters in pituitary MRI and is consistent with previously published radiomics and adenoma imaging research (24). All MRI examinations were performed within 1 week prior to surgery to ensure consistency and minimize temporal variability affecting radiomic feature stability.

### Radiomics feature extraction

Radiomic features were extracted from preoperative coronal-enhanced MRI sequences. First, the images were preprocessed, including normalization, registration, and denoising. Intensity normalization was performed on a per-subject basis by scaling the voxel intensities to zero mean and unit variance within each image volume. Rigid registration was applied using ANTs software, aligning each subject’s scan to a standardized anatomical template in MNI space to correct for head positioning differences. Denoising was performed using a non-local means filter to suppress random noise while preserving edge information. Then, regions of interest (ROI) were manually delineated using ITK-SNAP software (27). The ROIs were independently delineated by two clinical experts, each with over 10 years of experience in neuro-oncology. The segmentation was performed in a double-blind manner within the same week, and inter-observer agreement was assessed using the intraclass correlation coefficient (ICC), which is described in detail in a later section.

The segmentation boundaries were defined as follows: Superior boundary: limited by the optic chiasm or suprasellar cistern; Inferior boundary: extending to, but not including, the sphenoid sinus; Lateral boundary: restricted by the medial walls of the cavernous sinus; Anterior and posterior boundaries: defined by visible enhancing tumor margins. Segmentation was performed slice-by-slice along the lesion contour in the axial plane, with coronal and sagittal views referenced to improve boundary accuracy.

**Figure 2:**
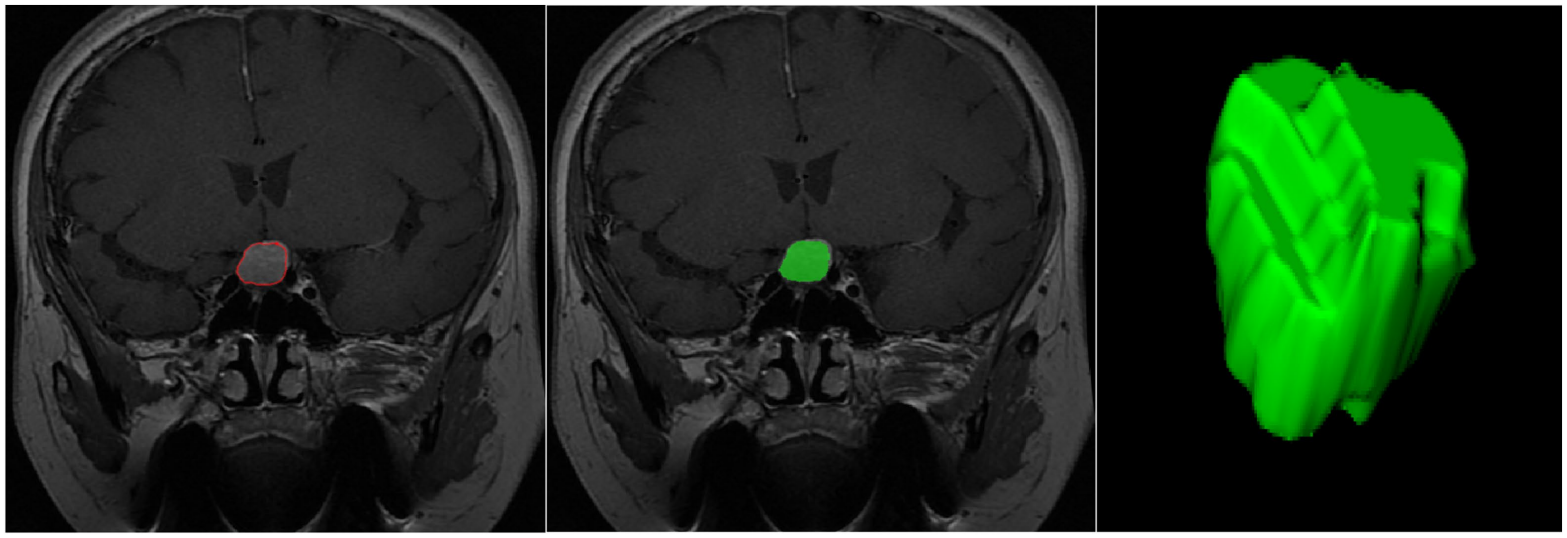
Schematic illustration of the ROI segmentation process.

Finally, features were extracted from the ROIs using the Pyradiomics package in Python (28), as illustrated in Figure 2. The extracted features included first-order statistical features (e.g., mean gray level, standard deviation), shape features, texture features (e.g., gray-level co-occurrence matrix, gray-level run-length matrix), and higher-order filtered features (e.g., wavelet-transformed features, log-sigma features). A total of 944 features were extracted for each patient. Specifically, 18 first-order features, 14 shape features, and 75 texture features were extracted [including gray-level co-occurrence matrix (GLCM), gray-level run-length matrix (GLRLM), gray-level size zone matrix (GLSZM), gray-level dependence matrix (GLDM), and neighboring gray-tone difference matrix (NGTDM)]. Additionally, 837 filtered features were generated using wavelet and Laplacian of Gaussian (LoG) filters. Wavelet features were computed across eight decomposition directions (e.g., LLL, LLH, LHL, LHH, etc.), and LoG features were calculated using sigma values of 1.0, 2.0, and 3.0.

### Histopathological and immunohistochemical assessment

All surgical specimens were subjected to routine histopathological examination. Hematoxylin and eosin (H&E) staining was initially performed to confirm the diagnosis of pituitary adenoma. Subsequently, immunohistochemical (IHC) staining was performed to evaluate p53 expression. Formalin-fixed, paraffin-embedded tumor tissue blocks were sectioned at a thickness of 4 μm and processed using an automated staining platform (Ventana Benchmark XT, Roche Diagnostics). A monoclonal mouse anti-human p53 primary antibody (clone DO-7, dilution 1:200, Dako/Agilent Technologies) was applied. The staining procedure included standardized antigen retrieval, incubation with the primary antibody, application of a HRP-conjugated secondary antibody, and visualization using a 3,3′-diaminobenzidine (DAB) chromogen. Known positive p53-expressing tissue samples and negative controls (absence of primary antibody) were included in each batch to ensure staining consistency and reliability.

The immunohistochemical evaluation was independently performed by two board-certified neuropathologists with over 10 years of diagnostic experience, respectively. Nuclear staining intensity and the percentage of positively stained tumor cells were recorded. In cases of disagreement, a joint consensus review was conducted to finalize the result.

Based on widely accepted pathological standards, tumors demonstrating ≥10% nuclear staining were classified as p53-overexpressed, while those with <10% nuclear positivity were categorized as non-overexpressed (29–31).

### Data splitting and feature reproducibility assessment

**Figure 3:**
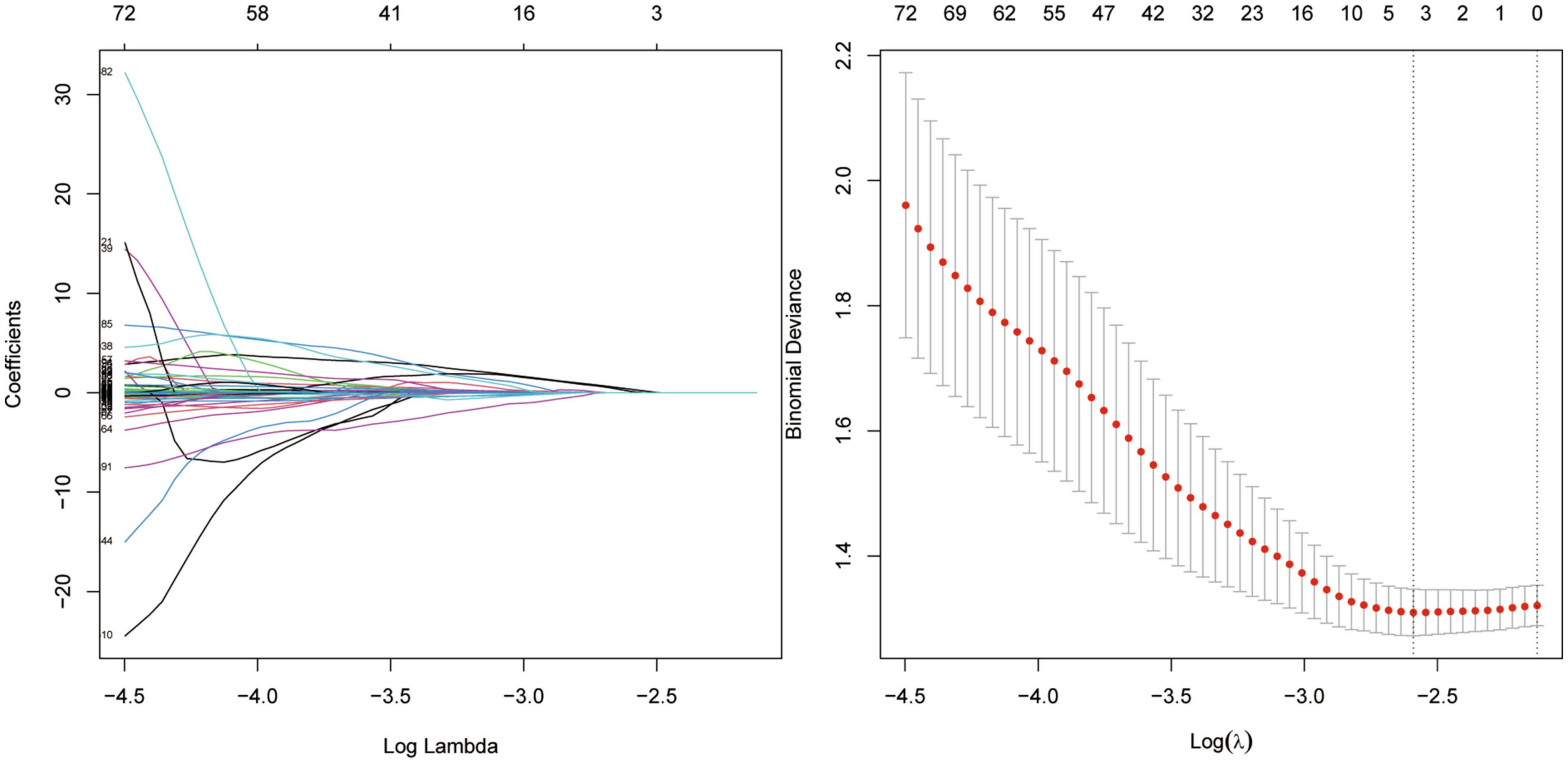
Selection of radiomic features using the LASSO method.

The dataset was randomly divided into a training set and a testing set in a 7:3 ratio. To ensure feature robustness and reduce overfitting, a two-step feature refinement process was performed, including reproducibility assessment using the Intraclass Correlation Coefficient (ICC) and subsequent dimensionality reduction using Least Absolute Shrinkage and Selection Operator (LASSO) regression (Figure 3).

For reproducibility evaluation, two clinical experts—each with over 10 years of experience in neuro-oncology imaging—independently delineated the ROIs. One expert completed segmentation for all 186 cases, while the second expert randomly selected and annotated 99 cases. Radiomic features were extracted separately from both sets of ROI masks, and the inter-observer agreement was assessed using the ICC. Features demonstrating good reproducibility (ICC ≥ 0.75) were retained for further modeling. A total of 364 stable features remained after this filtering step.

Subsequently, 10-fold cross-validation–guided LASSO regression was applied on the training set to reduce dimensionality and avoid overfitting. To prevent information leakage, feature selection—including ICC filtering and LASSO optimization—was performed only once on the training set and not repeated within each fold. This process yielded 4 optimal radiomic features, which were integrated to calculate the radiomics signature (Rad-score).

### Logistic regression analysis and nomogram model construction

Univariate logistic regression was performed for each clinical variable, and those with *p* < 0.05 were subsequently included in the multivariate logistic regression model to identify independent risk factors for p53 overexpression in PitNETs. By integrating the identified clinical risk factors with the radiomics score, a nomogram model was constructed using R software (32). The predictive performance of the model was evaluated on the testing set. Key evaluation metrics included the area under the receiver operating characteristic curve (AUC), precision-recall (PR) curve, calibration curve, and decision curve analysis (DCA).

### Statistical analysis

All statistical analyses were performed using R software (version 4.4.1) and Python (version 3.12.0) (33). The Mann–Whitney U test was used to analyze continuous variables, while Pearson’s χ^2^ test was applied for categorical variables. If small counts were present in contingency tables, Fisher’s exact test was used as an alternative to Pearson’s χ^2^ test. Univariate and multivariate logistic regression analyses were conducted to identify clinical risk factors, with a backward stepwise method used for variable selection. Variables with a *p* value < 0.05 in the univariate logistic regression were included in the multivariate analysis, and those with a *p* value < 0.05 in the multivariate regression were identified as independent risk factors.

## Results

### Baseline characteristics of patients

A total of 186 eligible patients with PitNETs were included in this study, comprising 91 males (48.92%) and 95 females (51.08%), with an average age of 53.5 years (range: 43.25–61.00 years). Postoperative immunohistochemical analysis showed that 118 patients (63.44%) were p53-positive, while 68 patients (36.56%) were p53-negative. Among all patients, 43 (23.12%) experienced pituitary apoplexy (PA), and 27 (14.52%) presented with cystic transformation (CT). Regarding Knosp grading, 112 patients (60.22%) had grades 0–2 adenomas, while 74 patients (39.78%) had grades 3–4. Based on tumor size, 84 cases (45.16%) were classified as giant adenomas, 97 cases (52.15%) as macroadenomas, and only 5 cases (2.69%) as microadenomas. Tumor invasion into the suprasellar (IS) region was observed in 85 cases (45.70%), with sphenoid sinus invasion (ISS) in 93 cases (50.00%). Furthermore, 112 cases (60.22%) involved encasement of more than three-quarters of the internal carotid artery (ECA), and 65 cases (34.95%) demonstrated cavernous sinus invasion (ICS). Laboratory test results showed that the average levels of key hormones were as follows: adrenocorticotropic hormone (ACTH) 30.00 (18.10, 39.10) pg./mL, cortisol 9.94 (7.57, 13.50) μg/dL, prolactin (PRL) 16.70 (9.21, 32.735) ng/mL, growth hormone (GH) 0.18 (0.08, 0.58) ng/mL, free triiodothyronine (FT3) 4.27 (3.59, 4.86) pmol/L, free thyroxine (FT4) 13.96 (11.68, 16.42) pmol/L, and thyroid-stimulating hormone (TSH) 1.70 (1.19, 2.75) mIU/L. The clinical baseline characteristics of the patients are summarized in Table 1.

**Table 1:** Baseline clinical characteristics of the patient cohort.

Variable	Categories	Train set (*n* = 130)		Test set (*n* = 56)		*P* value
		p53-negative (*n* = 48)	p53-positive (*n* = 82)	p53-negative (*n* = 20)	p53-positive (*n* = 36)	
Age (year)		55.50 (48.75, 66.00)	52.00 (39.50, 59.00)	56.50 (48.25, 66.25)	53.00 (39.50, 58.00)	0.004
Gender (%)	Male	24 (50.00)	42 (51.22)	12 (60.00)	13 (36.11)	0.497
	Female	24 (50.00)	40 (48.78)	8 (40.00)	23 (63.89)	
Knosp (%)	0–2	28 (58.33)	46 (56.10)	15 (75.00)	23 (63.89)	0.629
	3–4	20 (41.67)	36 (43.90)	5 (25.00)	13 (36.11)	
PA/CT (%)	No	34 (70.83)	49 (59.76)	12 (60.00)	21 (58.33)	0.513
	PA	9 (18.75)	22 (26.83)	5 (25.00)	7 (19.44)	
	CT	5 (10.42)	11 (13.41)	3 (15.00)	8 (22.22)	
Size (%)	Micro	4 (8.33)	0 (0.00)	0 (0.00)	1 (2.78)	0.108
	Macro	25 (52.08)	43 (52.44)	11 (55.00)	18 (50.00)	
	Giant	19 (39.58)	39 (47.56)	9 (45.00)	17 (47.22)	
IS (%)	No	21 (43.75)	49 (59.76)	9 (45.00)	22 (61.11)	0.050
	Yes	27 (56.25)	33 (40.24)	11 (55.00)	14 (38.89)	
ISS (%)	No	27 (56.25)	40 (48.78)	10 (50.00)	16 (44.44)	0.447
	Yes	21 (43.75)	42 (51.22)	10 (50.00)	20 (55.56)	
ECA (%)	No	18 (37.50)	33 (40.24)	11 (55.00)	12 (33.33)	0.653
	Yes	30 (62.50)	49 (59.76)	9 (45.00)	24 (66.67)	
ICS (%)	No	31 (64.58)	53 (64.63)	15 (75.00)	22 (61.11)	0.687
	Yes	17 (35.42)	29 (35.37)	5 (25.00)	14 (38.89)	
Acth (pg/mL)		30.20 (24.32, 44.45)	26.60 (14.70, 39.80)	22.95 (10.38, 36.45)	30.90 (25.43, 33.73)	0.586
Cortisol (ug/mL)		10.02 (8.37, 14.62)	10.02 (7.42, 13.57)	7.67 (3.11, 11.43)	10.10 (7.96, 12.80)	0.880
Prl (ng/mL)		15.87 (8.22, 27.99)	17.10 (10.05, 32.20)	13.32 (9.60, 30.30)	19.61 (11.49, 44.34)	0.186
Gh (ng/mL)		0.14 (0.09, 0.33)	0.18 (0.07, 0.75)	0.19 (0.10, 0.37)	0.23 (0.08, 0.79)	0.353
Ft3 (pmol/L)		4.24 (3.74, 4.54)	4.33 (3.54, 5.15)	4.14 (3.77, 4.75)	4.29 (3.58, 4.96)	0.227
Ft4 (pmol/L)		13.88 (12.21, 15.72)	13.93 (11.53, 16.34)	12.96 (11.07, 16.50)	14.37 (12.48, 17.48)	0.207
Tsh (mIU/L)		1.98 (1.28, 2.73)	1.81 (1.19, 2.83)	1.77 (1.08, 3.25)	1.56 (1.07, 2.09)	0.343

CT, Cystic Transformation; PA, Pituitary Apoplexy; ECA, Encasement of the Carotid Artery; ICS, Invasion of the Cavernous Sinus; IS, Invasion of the suprasellar; ISS, Invasion of the Sphenoid Sinus.

### Radiomics feature extraction and selection

From the preoperative MRI images of each patient, 944 radiomic features were extracted. After performing ICC and LASSO regression analyses, four features most strongly associated with p53 overexpression were retained: log.sigma.4.0.mm.3D_gldm_LargeDependenceHighGrayLevelEmphasis, wavelet. LLH_glcm_Correlation, wavelet. LHL_glcm_InverseVariance, and wavelet. HLL_ngtdm_Busyness. The process of feature selection is illustrated in Figure 2. Additionally, a radiomics score (Rad-score) was calculated to better assess the p53 status of patients. The formula for the Rad-score is as follows: Rad-score = (log.sigma.4.0.mm.3D_gldm_LargeDependenceHighGrayLevelEmphasis×0.00218) + (wavelet. LLH_glcm_Correlation×0.3224) + (wavelet. LHL_glcm_InverseVariance×0.10163) + (wavelet. HLL_ngtdm_Busyness×0.00021) − 0.36663.

This Rad-score provides a quantitative assessment to predict the p53 overexpression status more effectively. The violin plots in Supplementary Figure S1 illustrate the distribution of Rad-scores in the positive versus negative groups within both the training and testing datasets.

### Selection of clinical features

In the training set, univariate logistic regression analysis identified age, suprasellar invasion, and PRL levels as significantly associated with p53 overexpression (*p* < 0.05). Subsequently, the variables with statistical significance in the univariate analysis were included in a multivariate logistic regression analysis. Two independent clinical risk factors—age and suprasellar invasion—were ultimately identified (*p* < 0.05). The detailed results are presented in Table 2. The correlation heatmap in Supplementary Figure S2 illustrates the associations between radiomic features and clinically relevant classifications, including hormonal subtype, tumor size category, and invasion-related variables such as ECA, ICS, IS, and ISS.

**Table 2:** Univariate and multivariate logistic regression results for clinical risk factors.

Characteristics	Univariate analysis			Multivariate analysis		
	OR	95%CI	*P* value	OR	95%CI	*P* value
Gender	1.29	0.71–2.34	0.406			
Age	0.97	0.94–0.99	0.003	0.97	0.94–0.99	0.01
PA/CT	1.36	0.65–2.85	0.413			
Knosp	1.22	0.66–2.26	0.523			
Size	6.78	0.73–63.02	0.093			
IS	0.52	0.29–0.96	0.035	0.47	0.25–0.89	0.02
ISS	1.32	0.73–2.4	0.361			
ECA	1.21	0.66–2.21	0.545			
ICS	1.2	0.64–2.25	0.574			
Acth	1	0.98–1.01	0.744			
Cortisol	0.99	0.96–1.02	0.473			
Prl	1.01	1–1.02	0.046	1.01	1–1.02	0.198
Gh	1.03	0.99–1.08	0.137			
Ft3	1.24	0.94–1.65	0.13			
Ft4	1.06	0.99–1.13	0.075			
Tsh	1.01	0.96–1.08	0.632			

OR, odds ratio; CI, confidence interval.

### Model construction and evaluation

**Figure 4:**
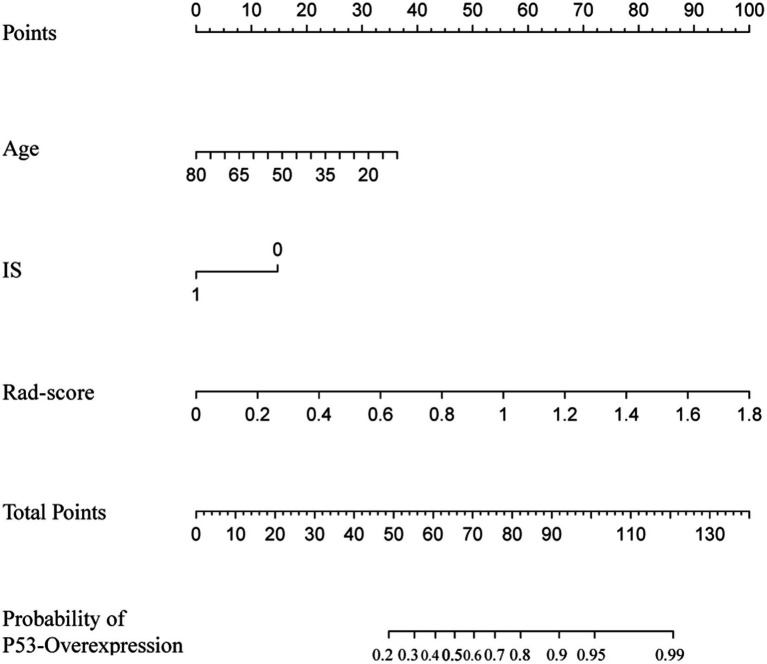
Nomogram model constructed for clinical applicability.

**Figure 5:**
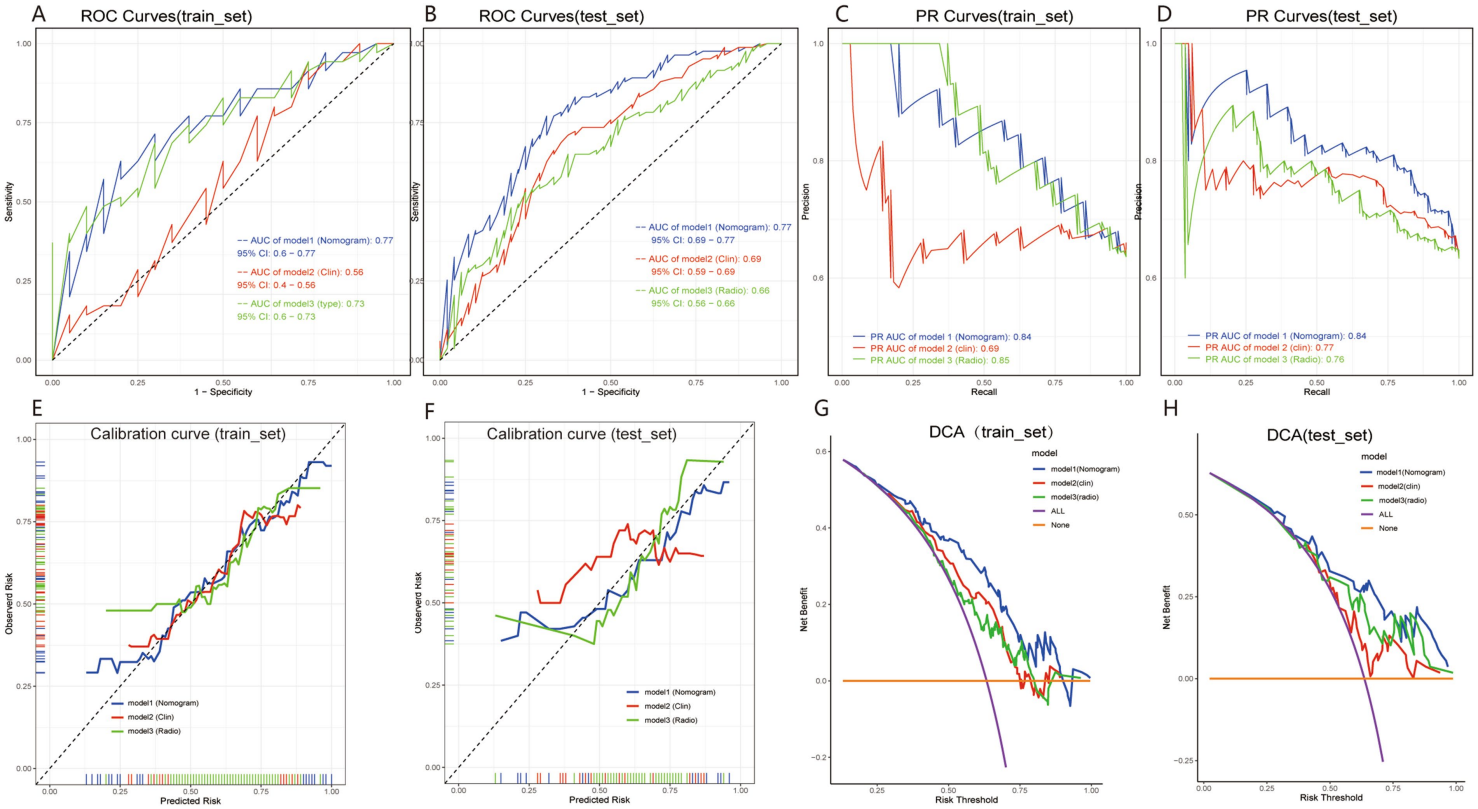
Performance evaluation of the radiomics model for preoperative prediction of P53 overexpression in pituitary adenoma patients. Panels **(A,B)** show ROC curves for the training and test sets, with AUC values illustrating the model’s discriminative power. Panels **(C,D)** present PR curves for each set. Calibration curves in panels **(E,F)** show alignment between predicted and observed probabilities, with the nomogram model closely following the ideal line. Panels **(G,H)** display DCA curves, demonstrating the clinical benefit of the nomogram model across various risk thresholds. These results highlight the model’s strong discrimination, calibration, and clinical utility.

The nomogram integrating the radiomics score with significant clinical predictors was constructed using multivariable logistic regression (Figure 4). In the training cohort, the model achieved an AUC of 0.77 (Figure 5A) and a PR value of 0.84 (Figure 5C). Similar performance was observed in the testing cohort, with an AUC of 0.77 (Figure 5B) and a PR value of 0.84 (Figure 5D).

Calibration curves demonstrated good agreement between predicted and observed probabilities in both the training and testing cohorts (Figures 5E,F). Decision curve analysis further showed a positive net benefit across a wide range of threshold probabilities in both cohorts (Figures 5G,H).

Overall, the integrated nomogram exhibited favorable discrimination, calibration, and clinical utility in predicting p53 overexpression.

## Discussion

This study developed and validated a nomogram model based on age, suprasellar invasion, and MRI-derived radiomics score to predict p53 overexpression in patients with PitNETs preoperatively. The results indicated that younger patients, those with suprasellar invasion, and patients with higher radiomics scores were more likely to exhibit p53 overexpression. The model demonstrated high predictive accuracy through multivariate integrative analysis, underscoring its potential for clinical application.

We found that younger patients received higher predictive scores in the nomogram model, consistent with findings from other tumor studies (34–36). Younger individuals often exhibit higher metabolic activity and tumor proliferation potential, which may lead to increased p53 overexpression. As a key regulator of the cell cycle and apoptosis, abnormal p53 overexpression is more common in highly active cells, increasing the likelihood of p53 overexpression in younger patients (37, 38). This finding suggests that age is an important factor to consider when predicting p53 overexpression preoperatively, with potential implications for clinical decision-making.

In this study, suprasellar invasion was closely associated with p53 overexpression. Suprasellar invasion often indicates aggressive tumor growth, reflecting higher biological activity and potentially stronger malignant features, including p53 overexpression (39). Suprasellar invasion signifies that the tumor has breached local boundaries and infiltrated surrounding structures, a biological behavior often linked to poor prognosis in various malignant tumors (40–42). Therefore, suprasellar invasion serves not only as an imaging marker of tumor aggressiveness but also as a crucial variable in predicting p53 overexpression preoperatively. Incorporating this feature into the nomogram model enables more accurate risk assessment for patients.

Radiomics enables the extraction of multidimensional quantitative descriptors from MRI, including shape features, texture metrics, and gray-level distribution patterns, which may serve as surrogates of intratumoral imaging heterogeneity (43–45). In the present study, higher radiomics scores were associated with an increased likelihood of p53 overexpression, suggesting that radiomics features may partially reflect biological behavior at the tissue level (46). However, this association should be interpreted cautiously, as the current findings do not establish a direct causal relationship but rather indicate a potential imaging–molecular link (47).

From a radiogenomic perspective, altered p53 expression has been associated with genomic instability, dysregulated apoptosis, and increased proliferative activity, which may subsequently influence tumor architecture and microenvironment (48, 49). These biological processes could manifest as heterogeneous cellularity, stromal remodeling, focal necrosis, cystic degeneration, or abnormal tumor vasculature. Texture-based radiomics features—such as entropy, gray-level non-uniformity, or spatial co-occurrence metrics—may reflect these underlying microstructural complexities, while higher-order features derived from wavelet or LoG filtering may further capture subtle spatial variability that is not visually appreciable on routine imaging (50).

Feature selection using LASSO regression retained shape- and texture-related parameters, with texture features demonstrating particularly strong discriminatory value for p53 expression. This trend aligns with the biological characteristics of PitNETs exhibiting abnormal p53 expression, which often display increased heterogeneity and proliferative irregularity (51). These findings indicate that radiomics may enable quantitative transformation of MRI-visible tissue characteristics into computational signatures capable of estimating molecular phenotypes in a non-invasive manner.

Taken together, the present analysis supports the potential utility of radiomics as a surrogate tool for predicting molecular biomarkers such as p53 expression. Nevertheless, these results remain exploratory, and future studies incorporating larger patient cohorts, multicenter datasets, multimodal imaging, and genomic or proteomic validation are necessary to further clarify the biological relevance and clinical applicability of this radiogenomic association.

This study developed a nomogram model that integrates age, suprasellar invasion, and radiomics score, providing clinicians with a preoperative tool for predicting p53 overexpression. The nomogram visually quantifies the contribution of each patient characteristic, illustrating the impact of different risk factors on p53 overexpression. In clinical practice, the model may assist preoperative evaluation by helping identify patients with a higher risk profile, potentially contributing to more refined surgical and postoperative treatment considerations. Moreover, the nomogram’s multivariate integrative analysis demonstrates its stability and practicality, offering high predictive accuracy and robustness, which supports personalized treatment strategies for PitNETs.

## Limitations and future directions

Despite the strong performance of the nomogram model, several limitations remain. First, this study is a single-center retrospective analysis with a limited sample size, necessitating validation in future multi-center studies to ensure the model’s generalizability. Second, the extraction of radiomic features depends on image quality and preprocessing methods, and further efforts are needed to standardize these processes. Future research should explore the relationship between other potential molecular markers and radiomic features, and incorporate more advanced machine learning techniques to enhance the predictive performance of the model.

## Conclusion

The nomogram model developed in this study, based on age, suprasellar invasion, and radiomics score, effectively predicts p53 overexpression in patients with PitNETs, providing valuable guidance for personalized preoperative treatment. By integrating radiomics features with clinical characteristics, the model demonstrates high predictive accuracy and strong potential for clinical application. Future efforts should focus on further validation and optimization of the model to promote its widespread use in clinical practice.

## Data Availability

The raw data supporting the conclusions of this article will be made available by the authors, without undue reservation.
