# Supplementary figures and images for: Preoperative prediction of p53 overexpression in pituitary neuroendocrine tumors using MRI radiomics
Source: Front Neurol. 2026 Jan 23;16:1693959. doi: 10.3389/fneur.2025.1693959 (PMC12876171; doi:10.3389/fneur.2025.1693959)

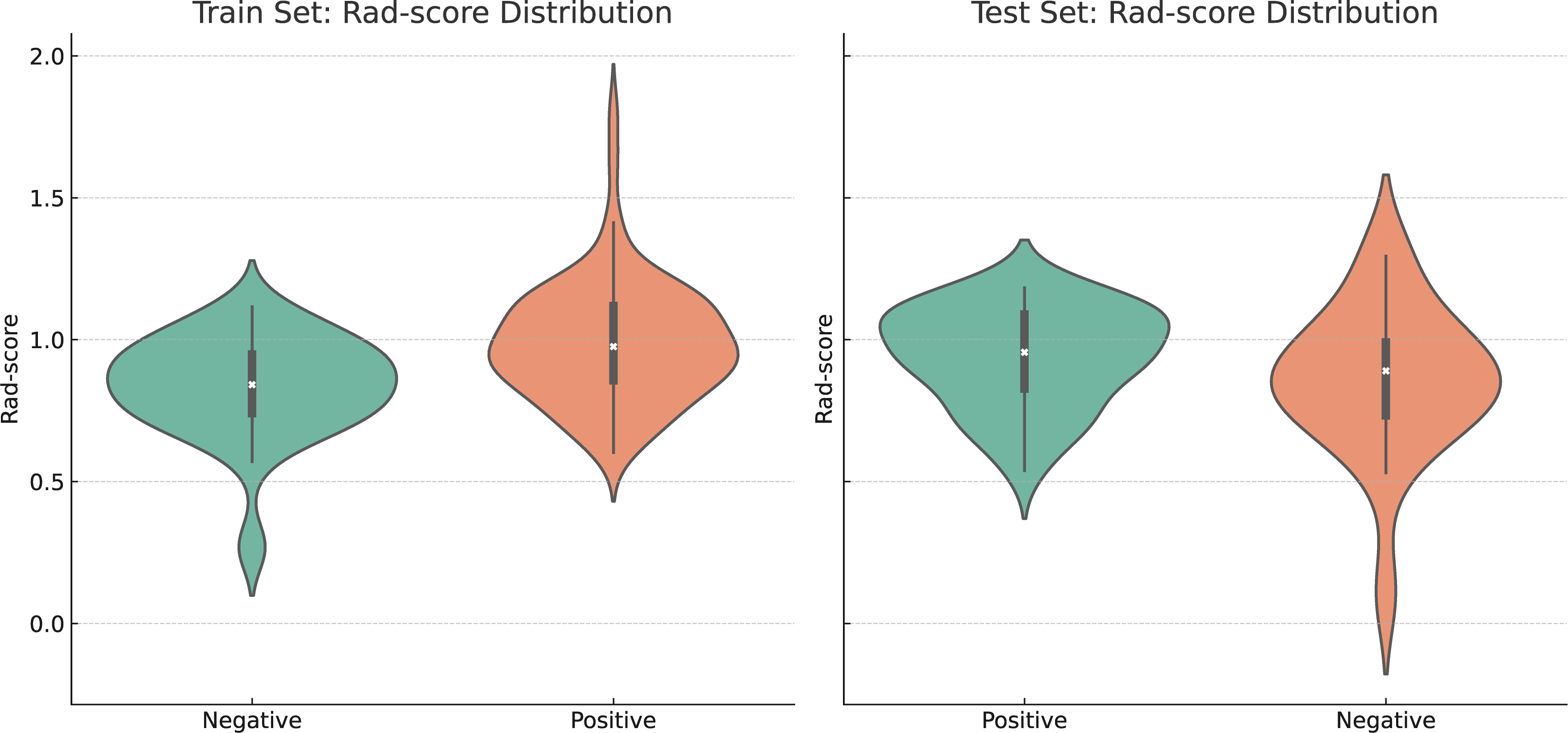

Supplement: Supplementary file 1 [file Image_1.tif]

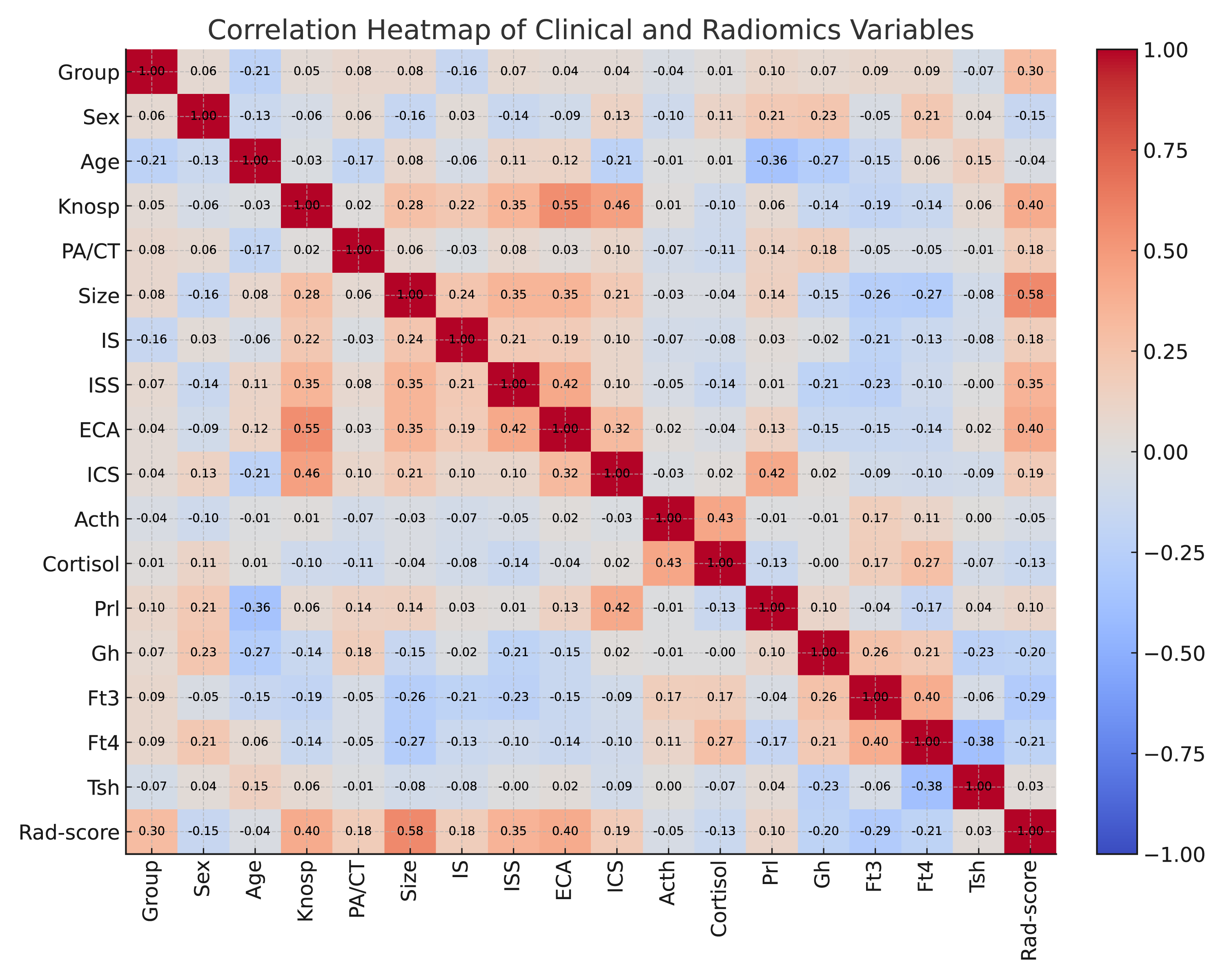

Supplement: Supplementary file 2 [file Image_2.tif]
